# Evaluation of the Differences between Axial and Double Oblique Measurements of the Ascending Aorta on Gated, Contrast-Enhanced Thoracic Computed Tomography Scans: A Technical Note

**DOI:** 10.1055/a-2537-5390

**Published:** 2025-04-22

**Authors:** Paul Stark, Eric Y. Chang

**Affiliations:** 1 Radiology Service, VA San Diego Healthcare System and University of California, San Diego, California

**Keywords:** CT, tubular ascending aorta, dimensions, axial, double oblique

## Abstract

**Background:**

The purpose of the study was to compare axial measurements of the ascending thoracic aorta on gated CT study studies with double oblique measurements and calculate the divergence between orthogonal axial and double oblique diameters of the tubular ascending aorta.

**Methods:**

Retrospective measurements of axial and double oblique diameters were obtained in 153 consecutive patients.

**Results:**

On average, the axial dimension exceeded the double oblique measurement.

**Conclusion:**

Our study endorsed the subtraction of 0.58 mm from the axial diameter in order to obtain the double oblique measurement.

Visualization of the thoracic aorta is routinely achieved with computed tomographic (CT) scanning.

Dilation of the ascending aorta is of particular concern in elderly patients with systemic hypertension, age-related degeneration of the aortic wall, or poststenotic dilation in aortic valvular stenosis. Bicuspid aortopathy and syndromic, hereditary conditions like Marfan's syndrome, Loes–Dietz syndrome, Turner syndrome, and Ehlers–Danlos Type IV have repercussions on the ascending aorta with propensity for dilation, aneurysm formation, and aortic dissection.

Routine measurements of the ascending aortic diameter are easily and regularly performed on routine axial CT scans. 1

Accurate measurements of the tubular ascending aorta require double oblique projections and an additional step in postprocessing and interpretation. 2 3

The purpose of this study was to compare axial measurements of the tubular ascending aorta with double oblique measurements and systematically calculate the divergence between orthogonal axial and double oblique diameters of the ascending aorta.

## Materials and Methods

**Fig. 1:**
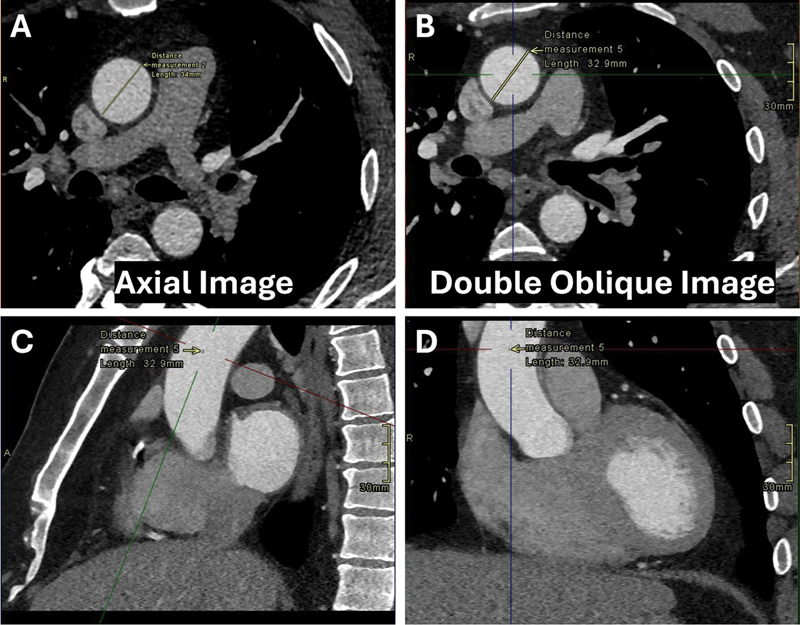
Comparison of axial ( **A** ) and double oblique planes ( **B** ) for measurement of the tubular ascending aortic diameter. The double oblique view is obtained by utilizing the center line in the axial source image and multiplanar reformation in the sagittal ( **C** ) and coronal ( **D** ) planes. In this example, the axial diameter measures 34 mm and the double oblique diameter measures 32.9 mm.

The institutional review board approved this Health Insurance Portability and Accountability Act-compliant study and waived informed consent. We retrospectively measured the diameter of the tubular ascending aorta in axial and double oblique planes in 153 consecutive patients examined with ECG-gated or ECG-triggered cardiac CT scans for coronary artery disease. We measured the aortic diameter at the level of the bifurcation of the main pulmonary artery in diastole at 75% of the R–R interval. Double oblique planes were generated by aligning the orthogonal planes with the long axis ( Fig. 1 ). The measurements were performed manually perpendicular to the direction of the flow of blood at an angle of 45 degrees to the center line (see image) and the aortic wall thickness was excluded. The measurements were performed in the same session by the first author (P.S.). The Kolmogorov–Smirnov test confirmed the data was normal and a paired samples *t* -test was used to determine if differences existed between the two groups. Pearson's correlations were performed. Simple linear regression was performed to determine the relationship between the variables. A *p* -value less than 0.05 was considered statistically significant. Analyses were performed using SPSS (Version 21.0 IBM SPSS Chicago, IL).

## Results

The mean diameter of the tubular ascending aorta in the axial plane in our population was 34.3 ± 4.6 mm (mean ± standard deviation). In the double oblique projection, the mean diameter of the tubular ascending aorta was 33.9 ± 4.4 mm. The differences between groups were statistically significant ( *p* = 0.004). The correlation between the two measurements was 0.96, i.e., close to 1 ( *p* = 0.001).

**Fig. 2:**
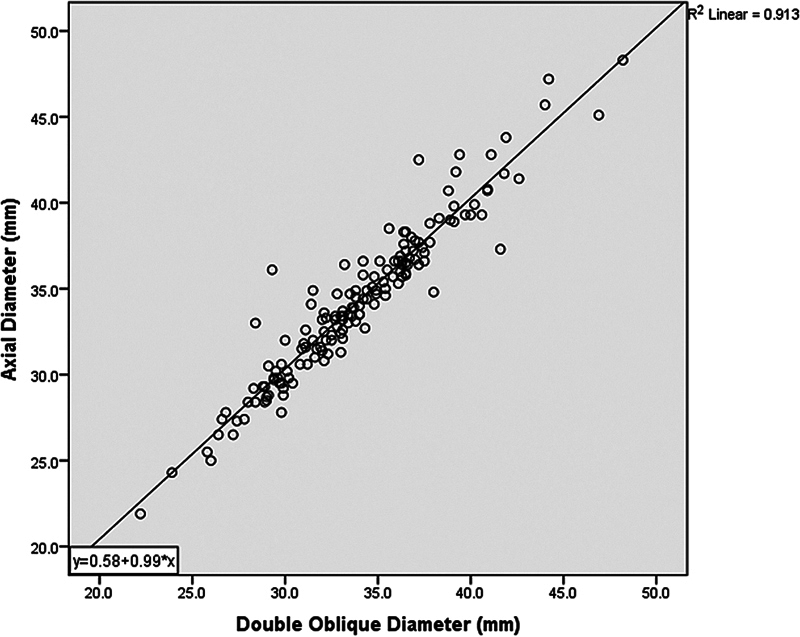
Scatter plot showing relationship between aortic diameter on the axial versus double oblique imaging planes. To convert from axial to double oblique, take the axial measurement, subtract 0.58 and divide that by 0.99.

Conversion of axial to double oblique measurements for the tubular ascending aorta can be obtained by subtracting 0.58 mm from the axial diameter ( Fig. 2 ).

## Discussion

The tubular ascending aorta is approximately 5 to 8 cm long and has a width of 30 to 40 mm or indexed to 21 mm/square meter of body surface area. 2 The normal aortic wall thickness measures 1 to 3 mm. 3

As part of the normal aging process, the ascending aorta width increases by 0.5 to 1 mm per year.

In adults, the aortic dimensions are related to body surface area, exercise, and workload. The aortic diameter varies also according to cardiac cycle and is 2 to 3 mm wider in systole. If the aortic wall is included in the measurement, it may increase the overall diameter by 2 to 6 mm. 3 We excluded the aortic wall thickness in our measurements. We selected the tubular ascending aorta because in our population it has the least degree of noncircularity.

A C-shaped ascending aorta may distort the axial measurements, which can exceed the true diameter measured perpendicularly to the aortic long axis. Thus, obliquity of the axial plane related to the aortic center line will introduce a moderate discrepancy of up to several millimeters and the axial measurement will overestimate the diameter in the double oblique plane.

Overall, for the majority of aortas devoid of excessive lengthening and little curvature convex to the right, the axial diameter and the dimensions obtained with double oblique measurements will differ very little. 3

As a proof of concept, we attempted to find a constant measure that could be subtracted from the axial diameter of the ascending aorta and yield the double oblique diameter of the ascending aorta, while saving time and avoiding the routine axial image conversion into a double oblique display.

Our results endorse the deduction of 0.58 mm from the axial diameter to yield the double oblique measurement of the tubular ascending aorta.
